# The Global Prevalence of *Neospora caninum* Infection in Sheep and Goats That Had an Abortion and Aborted Fetuses: A Systematic Review and Meta-Analysis

**DOI:** 10.3389/fvets.2022.870904

**Published:** 2022-04-26

**Authors:** Tooran Nayeri, Shahabeddin Sarvi, Mahmood Moosazadeh, Ahmad Daryani

**Affiliations:** ^1^Toxoplasmosis Research Center, Mazandaran University of Medical Sciences, Sari, Iran; ^2^Department of Parasitology, School of Medicine, Mazandaran University of Medical Sciences, Sari, Iran; ^3^Student Research Committee, Mazandaran University of Medical Sciences, Sari, Iran; ^4^Gastrointestinal Cancer Research Center, Non-communicable Diseases Institute, Mazandaran University of Medical Sciences, Sari, Iran

**Keywords:** *Neospora caninum*, sheep, goat, abortion, fetus, meta-analysis

## Abstract

*Neospora caninum* (*N. caninum*) can be a potential factor causing a significant rate of miscarriages in small ruminants (sheep and goats) worldwide. Therefore, the present study aimed to determine the global status of *N. caninum* in sheep and goats that had an abortion and aborted fetuses. Five English databases (PubMed, ScienceDirect, Web of Science, Scopus, and ProQuest) were searched for relevant scientific articles published from their inception until November 4, 2021. Finally, 21 studies conducted on sheep (1,671 aborted fetuses and 935 abortive sheep) and 10 studies on goats (130 aborted fetuses and 80 abortive goats) were included for the final meta-analysis. A random-effects meta-analysis model was used to estimate the pooled prevalence with 95% confidence intervals (CIs). Moreover, sensitivity analysis, publication bias test, and quality assessment were performed in this study. The pooled prevalence of *N. caninum* in aborted fetuses of sheep and goats globally was estimated to be 15% (95% CI: 9–21%) and 7% (95% CI: 2–12%) using molecular methods. Besides, the seroprevalence of *N. caninum* was estimated to be 17% for aborted fetuses of sheep. The overall prevalence rate of *N. caninum* infection in sheep that had an abortion was 3%. The present results show a relatively high prevalence of *N. caninum* infection in sheep that had an abortion and aborted fetuses compared to goats. Therefore, further studies using different diagnostic techniques to more accurately estimate the rate of infection in sheep and goats may help provide adequate control measures and strategies to reduce the rate of abortion in sheep and goats and reduce economic damage to the livestock industry. This study was registered at the International Prospective Register of Systematic Reviews (PROSPERO; code: CRD42020216694).

## Introduction

*Neospora caninum* (*N. caninum*), an apicomplexan protozoan, is globally distributed and imposes significant economic losses to producers and the livestock industry (1). Various stages of the parasite's life cycle (tachyzoite, tissue cyst, and oocyst) mainly involve ruminants as intermediate hosts and canines as definitive hosts (2). This parasite is transmitted horizontally and vertically in herds. Abortion, stillbirth, or the birth of an asymptomatic infected animal may result from a placental infection of the fetus (3). This parasite can persist in farms and herds for years, and congenital transmission, the main route of abortion caused by *N. caninum*, plays an essential role in this regard (4). Although cattle are the most crucial host for *N. caninum*, natural infections have been reported in other ruminants like sheep and goats (5, 6). The prevalence of *N. caninum* infection in sheep and goats varies significantly across continents and countries worldwide (7–14). These variations in seroprevalence may be related to specific characteristics of each region, such as climatic conditions, differences in the nutritional and health management of animals, using different techniques in serological diagnosis, sheep and goat populations, and different design of a study (15, 16). Based on the findings of the systematic review and meta-analysis studies, the estimated seroprevalence of *N. caninum* infection in sheep and goats worldwide was reported to be 12 and 5.99 % (16, 17). Sheep are usually grazing, so they are more at risk for pathogens near the ground than goats, which are generally browsers (18). Experimental inoculation of small ruminants with *N. caninum* during pregnancy creates conditions similar to those observed in cows (19). However, neosporosis's clinical, epidemiological, and economic importance in sheep and goats has not been fully understood yet due to the limited number of studies (7).

In many cases, the exact cause of the abortion cannot be determined because a wide range of factors may be involved. Nevertheless, infectious causes seem to be predominant in sheep and goats. As the diagnosis must be made in a specialized veterinary laboratory, a high percentage of abortions remain undiagnosed (20, 21). The economic losses of ruminant's reproductive failure caused by *N. caninum* infection worldwide are estimated at 1.3 billion dollars annually (22); therefore, its role in the abortion of sheep and goats should not be ignored. To diagnose *N. caninum* infection in aborted fetuses, the researchers have worked on diagnostic methods with different sensitivities and specificities, such as histopathology (23), immunohistochemistry (IHC) (24), serology (25), and polymerase chain reaction (PCR) (26). There are limited studies on the prevalence of *N. caninum* infection in the aborted fetuses of sheep and goats. However, there is no comprehensive research to collect and systematically analyze this domain. Therefore, short communication and cross-sectional studies involving the aborted fetuses of sheep and goats, along with sheep and goats that had an abortion at different ages, were included in the study. The results were evaluated and presented as the pooled prevalence with a 95% confidence interval (CIs). Considering the critical role of *N. caninum* infection in ruminants' abortion, this meta-analysis aimed to estimate its prevalence in the aborted fetuses of sheep and goats using histopathology, IHC, serology, and PCR methods in the world, along with its prevalence in sheep and goats that had an abortion.

## Methods

### Study Design and Protocol Registration

This systematic review and meta-analysis exactly followed the protocol suggested by the Preferred Reporting Items for Systematic Reviews and Meta-analyses guidelines (Supplementary Table 1) (27). The systematic review and meta-analysis protocol is described on the PROSPERO website (https://www.crd.york.ac.uk/prospero/) with the registration code CRD42020216694.

### Inclusion and Exclusion Criteria

This study included observational (cross-sectional and short communication) studies available in English that examined the prevalence of *N. caninum* infection in aborted fetuses of sheep and goats with different diagnostic techniques, including histopathology, IHC, serology, and PCR, as well as articles about the prevalence of *N. caninum* in sheep and goats that had an abortion. On the other hand, the review articles, systematic review and meta-analysis articles, case-control studies, experimental studies, dissertations, conference papers, and protocol articles, as well as articles investigating the prevalence of *N. caninum* in sheep and goats with a history of abortion (more than one abortion), were excluded from the present study.

### Information Sources and Search Strategy

Five English databases (PubMed, ScienceDirect, Web of Science, Scopus, and ProQuest) were searched for related studies from February 15, 1997, to November 4, 2021, using a combination of keywords (“*Neospora caninum*” OR neosporosis) AND (abortion OR miscarriage OR “reproductive failure” OR “fetal loss”) AND (livestock OR ruminant OR sheep OR Ovis OR ovine) for sheep and (“*Neospora caninum*” OR neosporosis) AND (abortion OR miscarriage OR “reproductive failure” OR “fetal loss”) AND (livestock OR ruminant OR goat OR caprine OR Capra) for goat (Supplementary Table 2). Searching different databases with these keywords was done independently. Publications retrieved in the independent search of original articles were imported into a single Endnote file (EndNote X9, Thomas Reuters, Philadelphia, PA, USA), and duplicates were excluded. No restrictions were defined for the year of the publication, and the search was limited to the English language. Additional articles were identified through a hand search of reference lists and contact with authors of the original studies.

### Study Selection

Two trained researchers (TN and SS) assessed all identified titles and abstracts carefully. They selected the studies based on these criteria: participants, exposure conditions/cases, preferred outcome (s), and study designs. Subsequently, they obtained the relevant articles and screened the full text independently. Discrepancies between the reviewers were resolved through discussion and consensus by the third author (AD).

### Quality Assessment

The modified version of the Newcastle-Ottawa Scale (NOS) checklist was implemented to assess the quality of included records (28). The articles that scored less than 3 on the scale were considered low quality; scores ranging from 3 to 5 were moderate quality, while scores ranging from 6 to 7 were deemed high quality.

### Data Extraction

Two reviewers (TN and SS) independently extracted the required information from the included articles using a standardized form. The data comprised the first author, publication year, place of study, type of samples, diagnostic methods, sample size (the number of examined animals), results of histopathology, IHC, serology, and PCR methods (number of positive animals). In this study, information about the serum of sheep and goats that had an abortion or serum of dam was extracted. In cases where both samples were presented in the study, only the maternal serum was analyzed. In the case of aborted fetuses, brain, heart, lung, kidney, liver, tongue, spleen, adrenal gland, thymus, skeletal muscle, rumen, abomasum, gastric content, and placenta samples were used for molecular analysis, and fetal serum and fetal fluids were used for serological analysis.

### Statistical Analysis

The present meta-analysis was carried out using Stata software (version 14; Stata Corp, College Station, TX, USA). Random-effects models were used to estimate the pooled prevalence rates with 95% confidence intervals (CIs). The I-squared test was used to assess the extent of variations among the independent studies. I-squared values <25%, between 25 and 50%, and >50% were defined as low, moderate, and high heterogeneity, respectively. If heterogeneity is high, subgroup analysis is performed to explore the causes of heterogeneity among the selected studies. In this study, subgroup analysis was conducted based on diagnostic methods. In addition, Begg's rank test, Egger's regression test, and funnel plot were applied to present the publication bias and small study effects. To perform the sensitivity analysis, first, an article was deleted, and then the impact of this omission on the overall result of the study was determined.

## Results

### Study Identification and Selection

**Figure 1:**
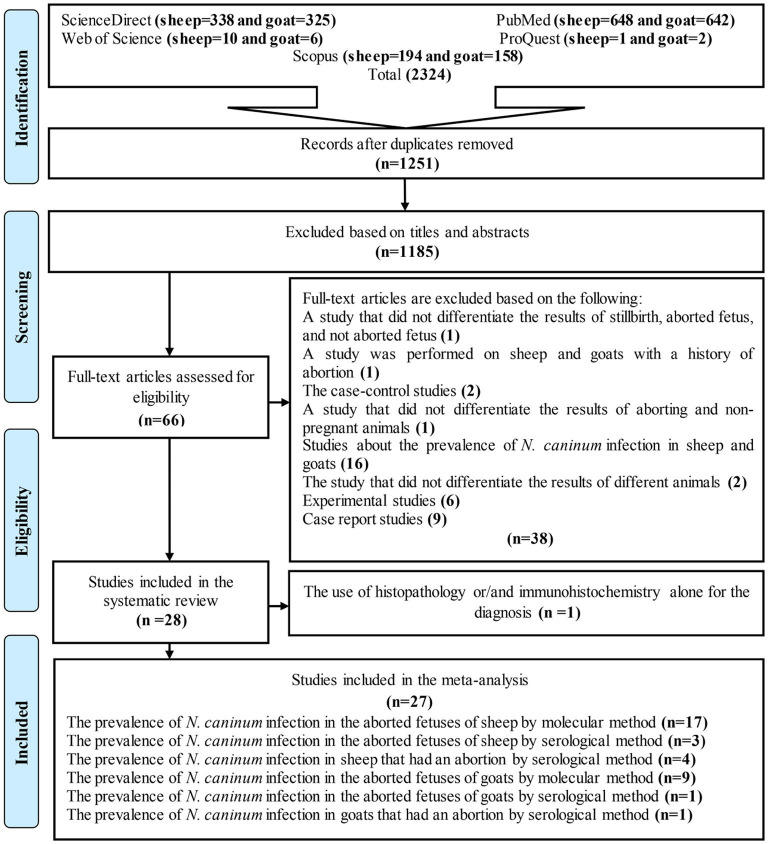
Flow diagram of the study design process.

Figure 1 is a flow chart showing the study selection process. In brief, 2,324 articles were found following the initial search of five databases. Duplicate articles (1,251 studies) were then removed using EndNote software. The articles were reviewed based on title and abstract in the next step, and 1,181 articles were excluded. Altogether, 70 studies were retained for further investigation and using their full texts. In some of these articles, the prevalence of *N. caninum* was studied in only one animal (sheep or goat), and others investigated two (sheep and goat). The final analyses included 22 studies on sheep and 12 studies on goats (Figure 1). Among 34 articles, seven examined the prevalence of *N. caninum* in both sheep and goats. Finally, 27 full texts were reviewed to determine the global status of *N. caninum* in sheep and goats that had an abortion and aborted fetuses (29–35). In this systematic review and meta-analysis, histopathology and IHC data were only presented, and no analysis was performed on them (Tables 1, 2).

**Table 1:** Description of the studies included for prevalence of *N. caninum* infection in sheep that had an abortion.

**Id**	**References**	**Place of study**	**Sample**	**Method**	**Sample size (*n*)**	**Serological results *n* (%)**	**Cut off**	**Molecular results *n* (%)**
1	Helmick et al. (36)	United Kingdom	Serum	IFA and ELISA	660	IFA: 3 (0.45) and ELISA: 28 (4.24)	IFA: ≥1/50 and ELISA: OD ≥0.38	–
2	Hässig et al. (37)	Switzerland	Serum	IFA	21	8 (38.09)	≥1:160	–
3	Špilovská et al. (38)	Slovak Republic	Serum	ELISA	184	4 (2.17)	–	–
4	Asadpour et al. (39)	Iran	Serum	ELISA	70	4 (5.71)	–	–

*IFA, indirect immunofluorescence assay; ELISA, enzyme-linked immunosorbent assay; and n, number*.

**Table 2:** Characteristics of the included studies for prevalence of *N. caninum* in the aborted fetuses of sheep.

**Id**	**References**	**Place of study**	**Sample**	**Methods**	**Sample size (n)**	**Serological results n (%)**	**Molecular results n (%)**	**Histopathology and IHC results n (%)**
1	Otter et al. (40)	United Kingdom	Pleural fluid	IFA	320	0 (0)	–	Histopathology: 21/290 (7.24) and IHC: 0/18 (0)
2	Hässig et al. (37)	Switzerland	Brain	Histopathology, IHC, and PCR	20	–	4 (20)	Histopathology: 4/20 (20) and IHC: 1/4 (25)
3	Hughes et al. (41)	United Kingdom	Brain	Nested-PCR	74	–	14 (18.91)	–
4	West et al. (42)	New Zealand	Fetal fluids	IFA	12	5 (41.66)	–	Histopathology: 4/9 (44.44)
5	Masala et al. (35)	Italy	Brain, skeletal muscle, liver, spleen, abomasum, and placenta	PCR	368	–	6 (1.63)	–
6	Moreno et al. (33)	Spain	Brain, lung, heart, liver, spleen, and kidney	Histopathology and nested-PCR	74	–	5 (6.8)	Histopathology: 8/74 (10.81)
7	Pinto et al. (43)	Brazil	Heart and brain	Histopathology and IHC	4	–	–	Histopathology: 2/2 (100) and IHC: 1/2 (50)
8	Asadpour et al. (39)	Iran	Brain, liver, gastric content, and placenta	PCR	70	–	6 (8.57)	–
9	González-Warleta et al. (5)	Spain	Brain, liver, heart, and lung	Histopathology, IHC, and PCR	4	–	4 (100)	Histopathology: 1/1 (100) and IHC: 1/1 (100)
10	Nunes et al. (29)	Brazil	Brain, liver, lung, kidney, and heart	Histopathology, IHC, and PCR	11	–	6 (54.54)	Histopathology: 0/11 (0) and IHC: 0/11 (0)
11	Schnydrig et al. (34)	Switzerland	Placenta and fetal abomasal content	Real-time PCR	7	–	0 (0)	–
12	Razmi & Naseri (44)	Iran	Brain	PCR	71	–	7 (9.85)	–
13	Díaz-Cao et al. (30)	Spain	Brain	Real-time PCR	11	–	0 (0)	–
14	Bartley et al. (45)	Scotland	Brain, heart, and placenta	Nested-PCR	119	–	0 (0)	–
15	Amouei et al. (31)	Iran	Brain	Nested-PCR	57	–	2 (3.5)	–
16	Al-Shaeli et al. (46)	Iraq	Placental, brain, heart, liver, lung, and thymus	Histopathology and PCR	42	–	6 (14.29)	Histopathology: 6/6 (100)
17	Meixner et al. (47)	Germany	Placenta	Real-time PCR	200	–	7 (3.5)	–
18	Khodadadi (48)	Iran	Placenta and brain	PCR	130	–	3 (2.3)	–
19	Salehi et al. (32)	Iran	Brain	Nested-PCR	51	–	8 (15.6)	–
20	Della Rosa et al. (25)	Argentina	Fetal cavity fluids, central nervous system, heart, lungs, liver, tongue, forelimb, and hindlimb muscles	Histopathology, IHC, IFA, and PCR	30	6 (20)	8 (26.66)	Histopathology: 8/30 (26.66) and IHC: 2/30 (6.66)

*IHC, immunohistochemistry; IFA, indirect immunofluorescence assay; PCR, polymerase chain reaction; Real-time PCR, real-time polymerase chain reaction; Nested-PCR, nested-polymerase chain reaction; and n, number*.

### General Characteristics of the Included Studies

The studies were from 14 countries located on four continents, including Europe (United Kingdom = 3, Spain = 3, Switzerland = 2, Slovak Republic = 1, Norway = 1, Italy = 1, Scotland = 1, and Germany = 1), Asia (Iran = 5 and Iraq = 1), North America (USA = 1), South America (Brazil = 4 and Argentina = 2), and Australia/Oceania (New Zealand = 1). The most common diagnostic tests in these studies were serology [enzyme-linked immunosorbent assay (ELISA) and indirect immunofluorescence assay (IFA)] and molecular [PCR and nested-polymerase chain reaction (nested-PCR)] tests (Tables 1–3). In addition, cross-sectional studies with a score of ≥3 were included in this study as acceptable quality articles based on the NOS checklist. The articles with a quality score <3 were excluded. Supplementary Table 3 illustrates the quality scores of the various eligible studies.

**Table 3:** Characteristics of the included studies for prevalence of *N. caninum* in the aborted fetuses of goats.

**Id**	**References**	**Place of study**	**Sample**	**Methods**	**Sample size (*n*)**	**Serological results *n* (%)**	**Molecular results *n* (%)**	**Histopathology and IHC results *n* (%)**
1	Engeland et al. (49)	Norway	Brain	Histopathology and IHC	23	–	–	Histopathology: 0/23 (0) and IHC: 0/23 (0)
2	Moeller Jr (20)	USA	Brain, heart, lung, kidney, liver, adrenal gland, thymus, skeletal muscle, rumen, abomasum, and placenta	Histopathology	211	–	–	Histopathology: 2/211 (22)
3	Masala et al. (35)	Italy	Brain, skeletal muscle, liver, spleen, abomasum, and placenta	PCR	31	–	2 (6.45)	–
4	Mesquita et al. (50)	Brazil	Brain and heart	Histopathology, IHC, and PCR	4	–	3 (75)	Histopathology: 2/4 (50) and IHC: 1/4 (25)
5	Costa et al. (51)	Brazil	Brain	Histopathology, IHC, and PCR	8	–	6 (75)	Histopathology: 6/8 (75) and IHC: 6/8 (75)
6	Unzaga et al.(52)	Argentina	Fetal fluids	IFA	25	5 (20)	–	–
7	Nunes et al. (29)	Brazil	Brain, liver, lung, kidney and heart	Histopathology, IHC, and PCR	6	–	3 (50)	Histopathology: 1/6 (16.66) and IHC: 1/6 (16.66)
8	Díaz-Cao et al. (30)	Spain	Brain	Real-time PCR	16	–	1 (6.25)	–
9	Amouei et al. (31)	Iran	Brain	Nested-PCR	4	–	0 (0)	–
10	Salehi et al. (32)	Iran	Brain	Nested-PCR	4	–	0 (0)	–
11	Moreno et al. (33)	Spain	Brain, lung, heart, liver, spleen and kidney	Histopathology and nested-PCR	26	–	3 (11.53)	Histopathology: 4/26 (15.38)
12	Schnydrig et al. (34)	Switzerland	Placenta and fetal abomasal content	Real-time PCR	6	–	1 (16.66)	–

*IHC, immunohistochemistry; IFA, indirect immunofluorescence assay; PCR, polymerase chain reaction; Real-time PCR, real-time polymerase chain reaction; Nested-PCR, nested-polymerase chain reaction; and n, number*.

### Prevalence of *N. caninum* Infection in Sheep That Had an Abortion

A total of 935 sheep that had an abortion were assessed, out of which 19 cases were positive for the antibodies against *N. caninum* using serological methods. Based on our results, a 3% (95% CI: −0–7%) seroprevalence was determined in sheep with an abortion. Results showed a strong heterogeneity (I^2^ = 83.67%, *p* = 0.00) among the selected studies (Supplementary Figure 1).

### Prevalence of *N. caninum* Infection in the Aborted Fetuses of Sheep

In this meta-analysis, 362 aborted sheep fetuses were examined for the antibodies against *N. caninum*, out of which 11 cases were positive using serological methods. The results of a random effect model showed that the seroprevalence of *N. caninum* in the aborted fetuses of sheep was 17% (95% CI: −5–39%). Supplementary Figure 2 shows a strong heterogeneity (I^2^ = 87.55%, *p* = 0.00) among the evaluated studies.

**Figure 2:**
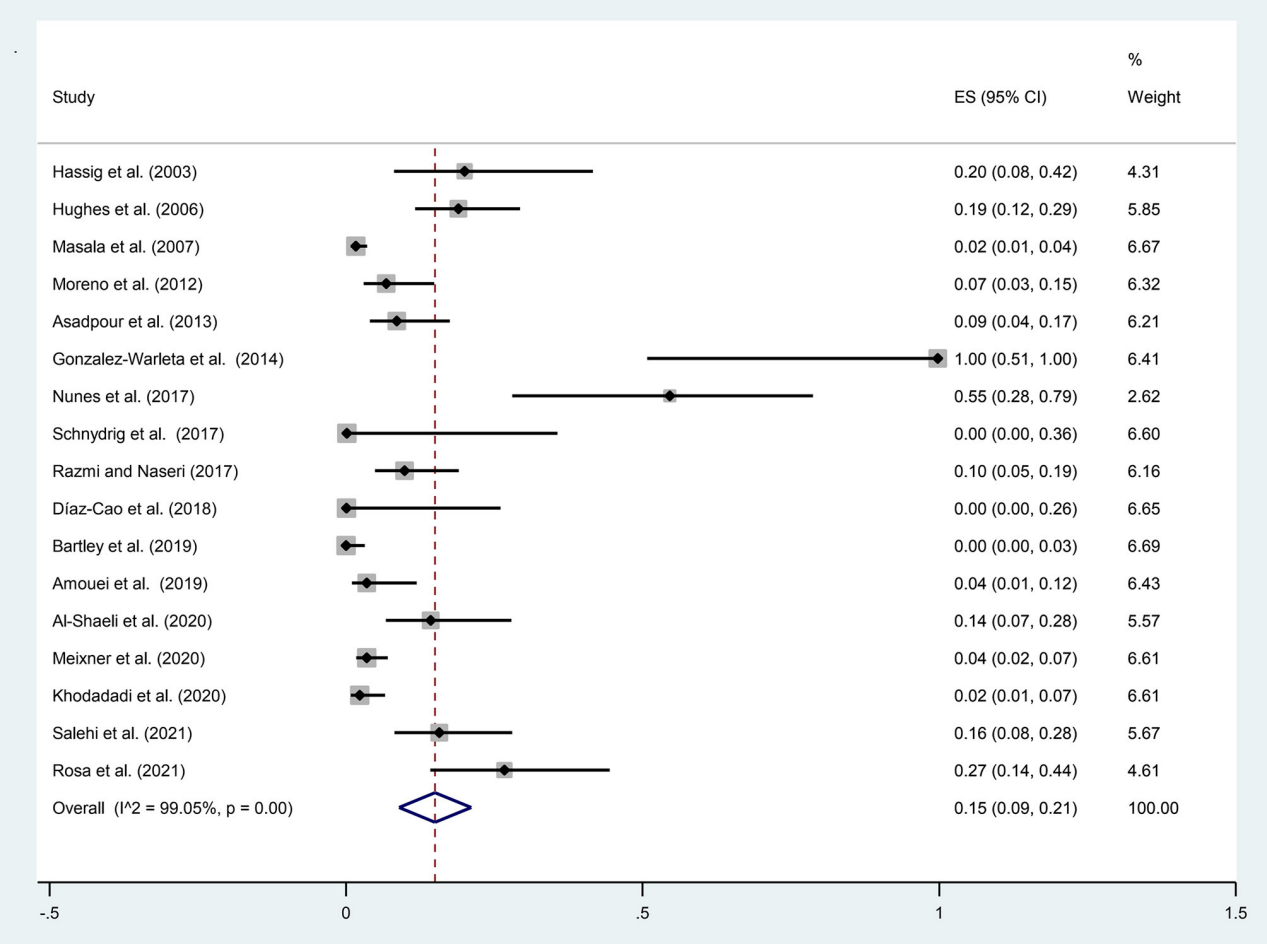
The prevalence of *N. caninum* infection in the aborted fetuses of sheep using molecular methods.

A total of 1,339 samples of the sheep aborted fetuses in 17 studies entered into the meta-analysis, out of which 86 cases were positive using molecular methods. According to Figure 2, the pooled global prevalence of *N. caninum* in the aborted fetuses of sheep was 15% (95% CI: 9–21%), with a high heterogeneity among studies (I^2^ = 99.05%, *p* = 0.00). Publication bias was observed using Egger's test (*p* = 0.002) in the included studies (Supplementary Figure 3). The results of the subgroup analysis revealed that the effect of assessment methods (PCR, nested PCR, and real-time PCR) on the heterogeneity of studies was not statistically significant (*p* = 0.108). Moreover, the sensitivity analysis confirmed the stability of the results of this study (Supplementary Figure 4). Nine studies used the histopathology method to evaluate lesions compatible with *N. caninum* in aborted fetuses of sheep. A total of 443 aborted fetuses were investigated by histopathology method; 50 aborted fetuses (11.29%) were positive for *N. caninum* infection. Furthermore, 66 aborted fetuses of sheep were examined by the IHC method in six articles; five aborted fetuses (7.58%) were positive for *N. caninum* infection.

### Prevalence of *N. caninum* Infection in Goats That Had an Abortion

In Norway, Engeland et al. (49) examined 80 blood samples from goats with abortion for the presence of antibodies against *N. caninum* using IFA; there were no positive cases.

### Prevalence of *N. caninum* Infection in the Aborted Fetuses of Goat

**Figure 3:**
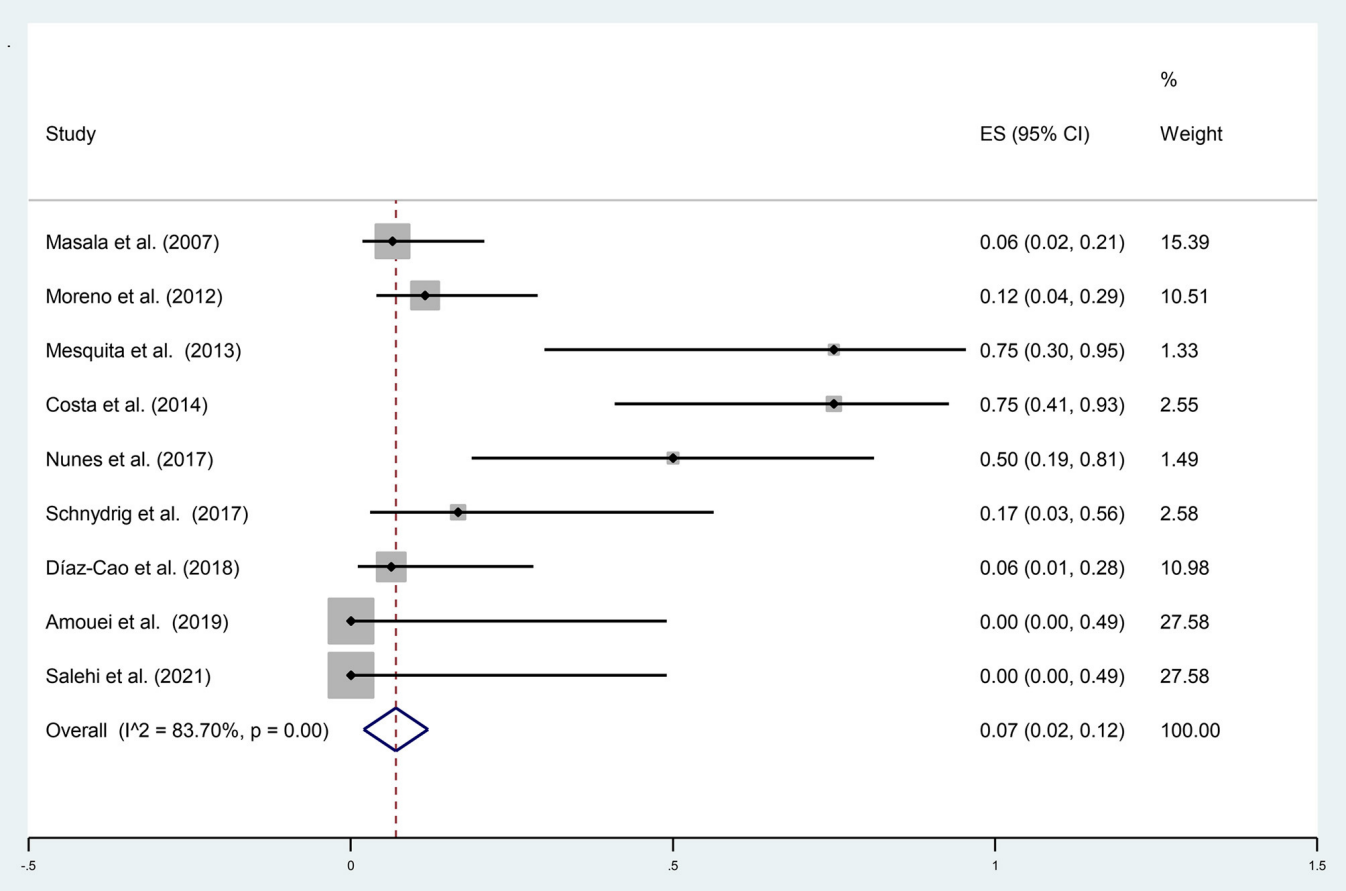
The prevalence of *N. caninum* infection in the aborted fetuses of goats using molecular methods.

A total of 105 samples of aborted goat fetuses were included in the present meta-analysis; 19 cases were positive using molecular methods. The pooled prevalence of *N. caninum* infection in aborted fetuses of goats in nine studies was estimated to be 7% (95% CI: 2–12%) based on molecular methods. Heterogeneity indicators have shown significant heterogeneity among the studies included in this meta-analysis (I^2^ = 83.70%, *p* = 0.00) by molecular techniques (Figure 3). Supplementary Figure 5 illustrated that Begg's rank test results showed no publication bias (*p* = 0.118). Based on the sensitivity analysis, the effect of excluding each study from the meta-analysis was not significant on the overall estimates (Supplementary Figure 6). A study performed by Unzaga et al. (52) estimated the seroprevalence of *N. caninum* infection in aborted goat fetuses in Argentina after examining 25 serum samples. The results showed that 20% (*n* = 5) of the animals were positive for *N. caninum*. Six studies used the histopathology method to diagnose *N. caninum* in aborted goat fetuses. A total of 278 aborted fetuses were examined; 15 (5.40%) were positive for *N. caninum* infection. Moreover, four articles employed the IHC method to investigate 41 aborted goat fetuses and found that 8 of them (19.51%) were positive for *N. caninum* infection. In most studies, the IHC method was performed only on positive samples using histopathology.

## Discussion

Studies have shown that non-infectious (nutritional, physical, toxic, and chemical) and infectious (viral, bacterial, fungal, and protozoan) agents may play a role in abortion (53–55). Among infectious agents, some bacterial (i.e., *Brucella* spp., *Chlamydia abortus, Coxiella burnetii, Leptospira* spp., *Listeria monocytogenes, Mycoplasma* spp., *Campylobacter jejuni, Campylobacter fetus* subsp. fetus, and *Salmonella Abortusovis*), viral (i.e., *Border disease virus* and *Bluetongue virus*), and protozoal (i.e., *T. gondii* and *N. caninum*) agents are the most common causes of pregnancy loss in sheep and goats (33, 56–58). Thus, we designed this systematic review and meta-analysis to explore the global prevalence of *N. caninum* infection in sheep and goats that had an abortion and aborted fetuses.

Histopathological examination in animals with neosporosis reveals extensive suppurative and non-suppurative meningoencephalitis (59). In addition, the incidence of various cellular and vascular lesions with glial reactions has been reported in the brain tissues of aborted fetuses containing focal or diffuse gliosis (39, 44, 59). Histopathological studies have shown that tissue cyst occurs mainly in central nervous system (39). The brain is the organ of choice for diagnosing neosporosis in aborted fetuses (5).

*N. caninum* infection is difficult to diagnose due to the ambiguous nature of the initial clinical signs and symptoms and the small number of parasites in the infected tissues (60). In addition, the clinical symptoms and pathological lesions are similar to those caused by *T. gondii*. *T. gondii* and *N. caninum* are cyst-forming apicomplexan parasites with similar clinical and pathologic features in sheep and goats. Hence, *N. caninum* may have been mistakenly identified as *T. gondii* in some studies (5, 33). However, histopathological evaluation of aborted fetal tissues is the basic method used to determine protozoan-induced abortion (61). In this attempt, the overall prevalence of *N. caninum* infection based on lesions histological was calculated at 11.29%, and 5.4% in the sheep and goats aborted fetuses. Identifying *T. gondii* and *N. caninum* tachyzoites and cysts is difficult in histological sections, especially in cases where the number of parasites is low. IHC is a technique that facilitates parasites visualization in tissues, although it is less sensitive (33, 62). IHC staining indicates that *N. caninum* has no cross-reaction with *T. gondii* or extra-intestinal coccidia (63). Various factors such as processing of tissue samples, the used detection system, primary antibody interaction, and type of tissue studied may affect IHC staining (47, 64). In most articles included in this meta-analysis, polyclonal antibodies were used in the IHC method. In this study, the overall prevalence of *N. caninum* infection in the aborted fetuses of sheep and goats using the IHC test was 7.58 and 19.51%, respectively.

Moreover, serological tests such as ELISA and molecular techniques such as PCR for the fetal serum and fetal fluids are prerequisites for confirming the diagnosis of an infectious agent causing a miscarriage diagnosed by histopathology (39). The pooled prevalence of the infection in the aborted sheep fetuses was 17% by serological methods. Besides, this systematic review process provided access to one study on the prevalence of *N. caninum* infection in aborted fetuses of goats. Immunoglobulin cannot cross the placenta in ruminants (cattle, sheep, and goats). Therefore, detecting parasite-specific antibodies in precolostrum sera indicates an active immune response. The fetus most likely synthesizes it against the parasite before birth (65). After birth, the ruminants gain passive immunity by eating immunoglobulin-rich colostrum from their dam. The half-life of passive immunoglobulins in small ruminants is short (25 days for IgG, 6 days for IgM, and 2 days for IgA) (66).

IFA and ELISA are the main serological tests used to diagnose neosporosis. IFA is the gold standard for the serological diagnosis of *N. caninum* infection and is very specific. Despite several common antigens, there are no cross-reactions between *N. caninum* and *T. gondii* (67). One of the gaps in these studies is that the articles included in this meta-analysis have not mentioned the antigen used in serological tests. However, indirect ELISA testing revealed cross-reaction with antibodies to *Sarcocystis* spp. and resulted in false-positive results (67). Positive results of serological tests indicate that the animal has been infected with *N. caninum*, whereas in the case of abortion, it does not provide a definitive diagnosis and analysis of aborted fetal tissues for the presence of specific lesions, tissue cysts, and tachyzoites that are necessary for confirming the diagnosis (68). Another main gap of the articles in this meta-analysis is that the sensitivity and specificity of different laboratory diagnostic methods have not been mentioned; it has a high impact on the results of this study. Therefore, it is recommended that the researchers use a reliable test with high sensitivity and specificity to achieve accurate results in different countries. This precise method can help interpret the studies' results correctly.

PCR, detecting specific DNA in limited samples, is one of the most accurate and widely used molecular methods to study the global prevalence of *N. caninum* infection in sheep and goats that had an abortion and aborted fetuses (69). In the current study, the pooled prevalence rate of infection is estimated to be 15% (95% CI: 9–21%) and 7% (95% CI: 2–12%) in the aborted fetuses of sheep and goats by molecular methods in the world. In addition, the overall prevalence rate of *N. caninum* infection in sheep that had an abortion was 3%. The search result led to just one article on the prevalence of *N. caninum* infection in goats that had an abortion.

The fetus's brain, heart, kidney, liver, and umbilical cord can be used for the molecular evaluation of aborted fetuses. In addition, due to this limitation that placental blood must be collected from the fresh placenta immediately after birth, the blood of the dam can be used instead of the placenta for the detection of *N. caninum* DNA (5, 69, 70). Molecular examination of the placenta is useful in diagnosing *N. caninum* infection in aborted fetuses, and placental infection indicates that the placenta has transmitted the infection to the fetal tissues (71). Although *N. caninum* is not a zoonotic disease, significant economic losses and animal welfare concerns caused by the parasite prompt researchers to evaluate essential factors involved in the infection (38). This parasite is considered a significant cause of abortion and reproductive failure in some dairy and beef herds and has a negative economic impact on the livestock industry (72). *N. caninum* has been reported sporadically as one of the causes of reproductive disorders in sheep (5, 7, 37, 73–76). Nevertheless, the role of *N. caninum* as a natural abortion agent in small ruminants needs to be further investigated because its experimental inoculation with *N. caninum* during pregnancy causes a situation very similar to that observed in cows (19).

It is noteworthy that heterogeneity was significant in all of these analyses (I^2^ > 50 %). It can be due to differences in geographical factors of each area, differences in the age of the ruminants in the studies, using different tissues to estimate prevalence in studies, and different diagnostic methods without similar specificities and sensitivities. However, a subgroup analysis was performed to investigate the effect of the diagnostic methods on heterogeneity. The findings indicated that the diagnostic methods had not affected the heterogeneity.

The main risk factors for neosporosis in ruminants that may be associated with abortion rates are the age of the animals, the size of the farm, the presence of dogs on farms, and the history of abortion. The role of age in *N. caninum* infection can be that females enter the reproductive life stage and contribute to the vertical transmission of the disease with the advancement of age. However, in horizontal transmission, the role of age in infection may be due to exposure to the sources of infection for a longer period in older animals (77, 78). An increase in the population density of the herd may increase the animal's contact with the infection source and different routes of *N. caninum* transmission in the environment (17).

Based on the findings of a systematic review and meta-analysis, there is no significant association between seropositivity in sheep with the occurrence of abortion and the presence of dogs on farms (17). Correspondingly, a unique meta-analysis has shown that the miscarriage risk in seropositive goats increases the probability of *N. caninum* seropositivity three times higher than in seronegative goats and the presence of dogs on farms (16). Finally, lack of assessment of risk factors such as type of sheepfold floor, rearing system, feeding, pasture area cultivated, worming, slaughter place of the animals, annual temperature, rainfall, evaluated tissue type, number of pregnancies, and type of birth in the included studies were the other gaps in the present meta-analysis.

As with the majority of studies, the design of the current study is subject to some limitations. Two limitations are the small number of included studies and insufficient data on the effects of risk factors for subgroup analysis. The other one is that the included studies did not mention the used antigens, sensitivity, and specificity of various diagnostic tests. Moreover, there is high heterogeneity, although it is a common finding for meta-analyses of prevalence studies. The last limitation is the lack of published articles on the global prevalence of *N. caninum* infection in sheep and goats that had an abortion and aborted fetuses in Africa.

## Conclusion

According to our data, the prevalence of *N. caninum* infection in sheep that had an abortion and aborted fetuses is relatively higher than in goats. The data presented in the current systematic review can be helpful for veterinarians by informing them about the epidemiology of *N. caninum* infection in sheep and goats around the world and the potential risk of this infection in abortion. Therefore, veterinarians should pay attention to this disease and take the necessary control measures to reduce the economic losses. However, further studies are imperative to better perceive the prevalence of *N. caninum* infection in sheep and goats that have had an abortion. Furthermore, other intensive studies can determine the role of this parasite in the etiology of abortion.

## Data Availability Statement

The original contributions presented in the study are included in the article/Supplementary Material, further inquiries can be directed to the corresponding author/s.

## Author Contributions

AD conceived and designed the study. SS critically revised the manuscript. TN searched the literature, extracted the data and wrote the manuscript. MM analyzed and interpreted the data. All authors have read and approved the final manuscript.

## Conflict of Interest

The authors declare that the research was conducted in the absence of any commercial or financial relationships that could be construed as a potential conflict of interest.

## Publisher's Note

All claims expressed in this article are solely those of the authors and do not necessarily represent those of their affiliated organizations, or those of the publisher, the editors and the reviewers. Any product that may be evaluated in this article, or claim that may be made by its manufacturer, is not guaranteed or endorsed by the publisher.
