# Supplementary figures and images for: The Global Prevalence of Neospora caninum Infection in Sheep and Goats That Had an Abortion and Aborted Fetuses: A Systematic Review and Meta-Analysis
Source: Front Vet Sci. 2022 Apr 26;9:870904. doi: 10.3389/fvets.2022.870904 (PMC9090472; doi:10.3389/fvets.2022.870904)

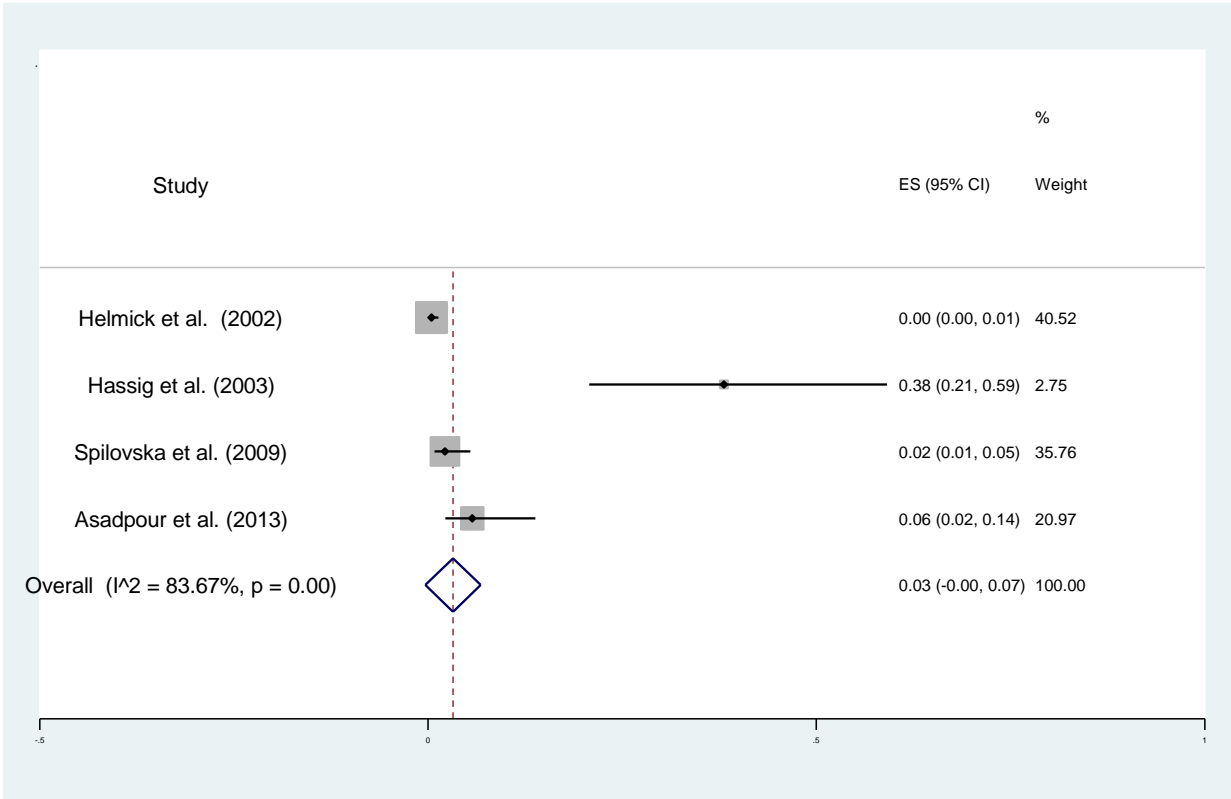

**Supplementary FIGURE 1** | The seroprevalence of *N. caninum* infection in sheep that had an abortion.

Supplement: Supplementary file 1 [file Image_1.pdf]
