# Supplementary material for: The Global Prevalence of Neospora caninum Infection in Sheep and Goats That Had an Abortion and Aborted Fetuses: A Systematic Review and Meta-Analysis
Source: Front Vet Sci. 2022 Apr 26;9:870904. doi: 10.3389/fvets.2022.870904 (PMC9090472; doi:10.3389/fvets.2022.870904)
Supplement: Supplementary file 3 [file Image_3.pdf]

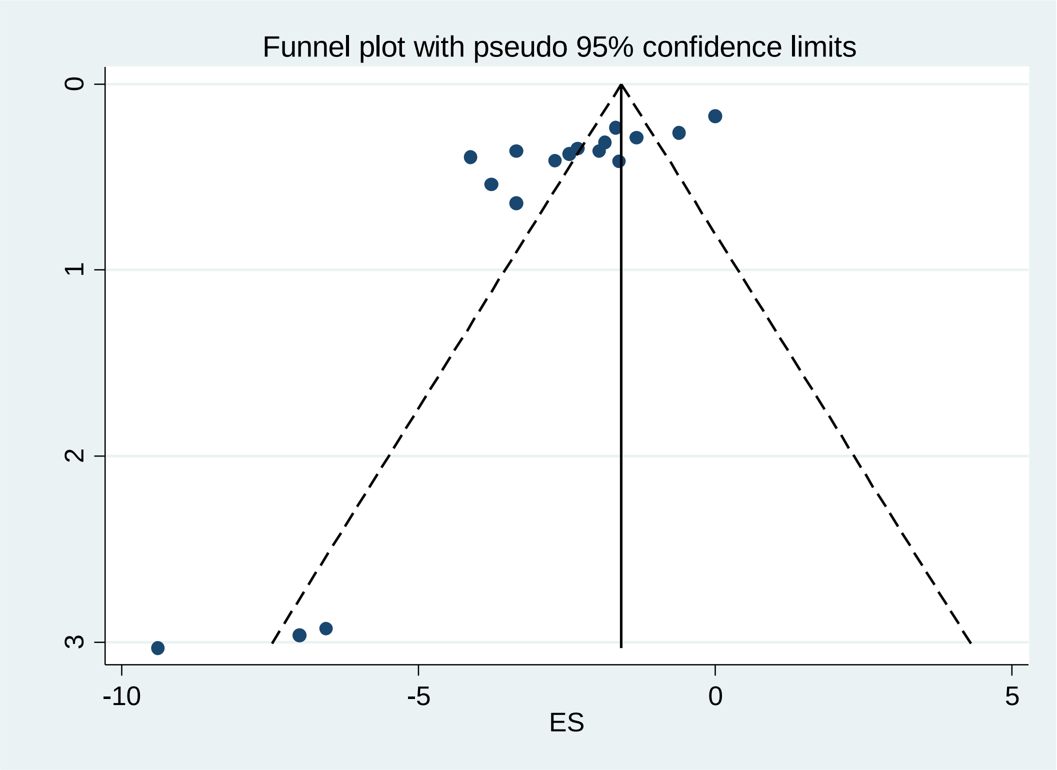

Supplementary FIGURE 3 | Funnel plot to detect publication bias in studies showing the prevalence of *N. caninum* infection in the aborted fetuses of sheep using molecular methods.
