# Supplementary material for: The Global Prevalence of Neospora caninum Infection in Sheep and Goats That Had an Abortion and Aborted Fetuses: A Systematic Review and Meta-Analysis
Source: Front Vet Sci. 2022 Apr 26;9:870904. doi: 10.3389/fvets.2022.870904 (PMC9090472; doi:10.3389/fvets.2022.870904)
Supplement: Supplementary file 8 [file Table_2.DOCX]

**Search strategy for sheep**

Supplementary Table 1. Search strategy in PubMed.

| Search | Search terms and combinations |
| --- | --- |
| 1 | (“*Neospora caninum*” OR neosporosis) |
| 2 | (abortion OR miscarriage OR “reproductive failure” OR “fetal loss”) |
| 3 | (livestock OR ruminant OR sheep OR ovis OR ovine) |
| 4 | #1 AND #2 AND #3 |
| 5 | Filters: Full text, Journal article, English language |

Supplementary Table 2. Search strategy in ScienceDirect.

| Search | Search terms and combinations |
| --- | --- |
| 1 | (“*Neospora caninum*” OR neosporosis) |
| 2 | (abortion OR miscarriage OR “reproductive failure” OR “fetal loss”) |
| 3 | (livestock OR ruminant OR sheep OR ovis OR ovine) |
| 4 | #1 AND #2 AND #3 |
| 5 | Filters: Research articles |

Supplementary Table 3. Search strategy in Scopus.

| Search | Search terms and combinations |
| --- | --- |
| 1 | (“*Neospora caninum*” OR neosporosis) |
| 2 | (abortion OR miscarriage OR “reproductive failure” OR “fetal loss”) |
| 3 | (livestock OR ruminant OR sheep OR ovis OR ovine) |
| 4 | #1 AND #2 AND #3 |
| 5 | Filters: All fields, Date range: Published in all years, Source type: articles, Language: English language |

Supplementary Table 4. Search strategy in ProQuest.

| Search | Search terms and combinations |
| --- | --- |
| 1 | (“*Neospora caninum*” OR neosporosis) |
| 2 | (abortion OR miscarriage OR “reproductive failure” OR “fetal loss”) |
| 3 | (livestock OR ruminant OR sheep OR ovis OR ovine) |
| 4 | #1 AND #2 AND #3 |
| 5 | Filters: Full text, Publication data: All data, Source type: Scholarly Journals, Document type: Article, Language: English language |

Supplementary Table 5. Search strategy in Web of Science.

| Search | Search terms and combinations |
| --- | --- |
| 1 | (“*Neospora caninum*” OR neosporosis) |
| 2 | (abortion OR miscarriage OR “reproductive failure” OR “fetal loss”) |
| 3 | (livestock OR ruminant OR sheep OR ovis OR ovine) |
| 4 | #1 AND #2 AND #3 |
| 5 | Filters: Topic, Document type: Article, Language: English language, Publication data: All data |

**Search strategy for goat**

Supplementary Table 1. Search strategy in PubMed.

| Search | Search terms and combinations |
| --- | --- |
| 1 | (“*Neospora caninum*” OR neosporosis) |
| 2 | (abortion OR miscarriage OR “reproductive failure” OR “fetal loss”) |
| 3 | (livestock OR ruminant OR goat OR caprine OR capra) |
| 4 | #1 AND #2 AND #3 |
| 5 | Filters: Full text, Journal article, English language |

Supplementary Table 2. Search strategy in ScienceDirect.

| Search | Search terms and combinations |
| --- | --- |
| 1 | (“*Neospora caninum*” OR neosporosis) |
| 2 | (abortion OR miscarriage OR “reproductive failure” OR “fetal loss”) |
| 3 | (livestock OR ruminant OR goat OR caprine OR capra) |
| 4 | #1 AND #2 AND #3 |
| 5 | Filters: Research articles |

Supplementary Table 3. Search strategy in Scopus.

| Search | Search terms and combinations |
| --- | --- |
| 1 | (“*Neospora caninum*” OR neosporosis) |
| 2 | (abortion OR miscarriage OR “reproductive failure” OR “fetal loss”) |
| 3 | (livestock OR ruminant OR goat OR caprine OR capra) |
| 4 | #1 AND #2 AND #3 |
| 5 | Filters: All fields, Date range: Published in all years, Source type: articles, Language: English language |

Supplementary Table 4. Search strategy in ProQuest.

| Search | Search terms and combinations |
| --- | --- |
| 1 | (“*Neospora caninum*” OR neosporosis) |
| 2 | (abortion OR miscarriage OR “reproductive failure” OR “fetal loss”) |
| 3 | (livestock OR ruminant OR goat OR caprine OR capra) |
| 4 | #1 AND #2 AND #3 |
| 5 | Filters: Full text, Publication data: All data, Source type: Scholarly Journals, Document type: Article, Language: English language |

Supplementary Table 5. Search strategy in Web of Science.

| Search | Search terms and combinations |
| --- | --- |
| 1 | (“*Neospora caninum*” OR neosporosis) |
| 2 | (abortion OR miscarriage OR “reproductive failure” OR “fetal loss”) |
| 3 | (livestock OR ruminant OR goat OR caprine OR capra) |
| 4 | #1 AND #2 AND #3 |
| 5 | Filters: Topic, Document type: Article, Language: English language, Publication data: All data |
