# Supplementary material for: The Global Prevalence of Neospora caninum Infection in Sheep and Goats That Had an Abortion and Aborted Fetuses: A Systematic Review and Meta-Analysis
Source: Front Vet Sci. 2022 Apr 26;9:870904. doi: 10.3389/fvets.2022.870904 (PMC9090472; doi:10.3389/fvets.2022.870904)
Supplement: Supplementary file 9 [file Table_3.DOCX]

**Supplementary Table 3 |** Quality assessment of included cross-sectional studies based on the Newcastle-Ottawa Scale.

| Id | First author | Publication year | Place of study | Type of study | Selection (3 points) | Comparability (2 points) | Outcome (3 points) | Total (scores) |
| --- | --- | --- | --- | --- | --- | --- | --- | --- |
| 1 | Otter *et al*. | 1997 | United Kingdom | Cross-sectional | 2 | 0 | 1 | 3 |
| 2 | Engeland *et al*. | 1998 | Norway | Cross-sectional | 2 | 0 | 2 | 4 |
| 3 | Moeller *et al*. | 2001 | USA | Cross-sectional | 2 | 0 | 1 | 3 |
| 4 | Helmick *et al*. | 2002 | United Kingdom | Cross-sectional | 2 | 0 | 1 | 3 |
| 5 | Hassig *et al*. | 2003 | Switzerland | Cross-sectional | 2 | 0 | 1 | 3 |
| 6 | Hughes *et al*. | 2006 | United Kingdom | Cross-sectional | 2 | 0 | 1 | 3 |
| 7 | West *et al*. | 2006 | New Zealand | Cross-sectional | 2 | 0 | 1 | 3 |
| 8 | Masala *et al*. | 2007 | Italy | Cross-sectional | 2 | 0 | 2 | 4 |
| 9 | Spilovska *et al*. | 2009 | Slovak Republic | Cross-sectional | 2 | 0 | 1 | 3 |
| 10 | Moreno *et al*. | 2012 | Spain | Cross-sectional | 2 | 0 | 1 | 3 |
| 11 | Pinto *et al*. | 2012 | Brazil | Cross-sectional | 2 | 0 | 1 | 3 |
| 12 | Asadpour *et al*. | 2013 | Iran | Cross-sectional | 2 | 0 | 2 | 4 |
| 13 | Mesquita *et al*. | 2013 | Brazil | Cross-sectional | 3 | 0 | 2 | 5 |
| 14 | Gonzalez-Warleta *et al*. | 2014 | Spain | Cross-sectional | 3 | 0 | 2 | 5 |
| 15 | Costa *et al*. | 2014 | Brazil | Cross-sectional | 2 | 0 | 1 | 3 |
| 16 | Unzaga *et al*. | 2014 | Argentina | Cross-sectional | 3 | 1 | 1 | 5 |
| 17 | Nunes *et al*. | 2017 | Brazil | Cross-sectional | 2 | 0 | 1 | 3 |
| 18 | Schnydrig *et al*. | 2017 | Switzerland | Cross-sectional | 2 | 0 | 2 | 4 |
| 19 | Razmi and Naseri | 2017 | Iran | Cross-sectional | 3 | 1 | 1 | 5 |
| 20 | Díaz-Cao *et al*. | 2018 | Spain | Cross-sectional | 2 | 0 | 1 | 3 |
| 21 | Bartley *et al*. | 2019 | Scotland | Cross-sectional | 2 | 0 | 2 | 4 |
| 22 | Amouei *et al*. | 2019 | Iran | Cross-sectional | 2 | 0 | 2 | 4 |
| 23 | Al-Shaeli *et al*. | 2020 | Iraq | Cross-sectional | 3 | 2 | 2 | 7 |
| 24 | Meixner *et al*. | 2020 | Germany | Cross-sectional | 2 | 0 | 2 | 4 |
| 25 | Khodadadi *et al*. | 2020 | Iran | Cross-sectional | 3 | 2 | 2 | 6 |
| 26 | Salehi *et al*. | 2021 | Iran | Cross-sectional | 2 | 0 | 2 | 4 |
| 27 | Rosa *et al*. | 2021 | Argentina | Cross-sectional | 2 | 0 | 1 | 3 |
